# Gut microbiota alternation under the intestinal epithelium-specific knockout of mouse *Piga* gene

**DOI:** 10.1038/s41598-022-15150-5

**Published:** 2022-06-25

**Authors:** Aditi Jangid, Shinji Fukuda, Masahide Seki, Yutaka Suzuki, Todd D. Taylor, Hiroshi Ohno, Tulika Prakash

**Affiliations:** 1 BioX Centre and School of Basic Sciences, Indian Institute of Technology Mandi, Kamand, Mandi, Himachal Pradesh 175005 India; 2 Institute for Advanced Biosciences, Keio University, Tsuruoka, Yamagata, 997-0052 Japan; 3 Laboratory for Intestinal Ecosystem, RIKEN Center for Integrative Medical Sciences, Yokohama, Kanagawa 230-0045 Japan; 4 Intestinal Microbiota Project, Kanagawa Institute of Industrial Science and Technology, Kawasaki, Kanagawa 210-0821 Japan; 5 Transborder Medical Research Center, University of Tsukuba, Tsukuba, Ibaraki 305-8575 Japan; 6 Department of Computational Biology and Medical Sciences, The University of Tokyo, 5-1-5, Kashiwanoha, Kashiwa Chiba, 277-8562 Japan; 7 Laboratory for Microbiome Sciences, RIKEN Center for Integrative Medical Sciences, Tsurumi-ku, Yokohama, Kanagawa 230-0045 Japan

**Keywords:** Computational biology and bioinformatics, Immunology

## Abstract

Crosstalk between the gut microbiota and intestinal epithelium shapes the gut environment and profoundly influences the intestinal immune homeostasis. Glycosylphosphatidylinositol anchored proteins (GPI – APs) contribute to a variety of gut-associated immune functions, including microbial surveillance and defense, and epithelial cell polarity. Properly polarised epithelial cells are essential for the establishment of the barrier function of gut epithelia. The *Piga* gene is one among seven genes that encode for an enzyme which is involved in the first step of GPI-anchor biosynthesis. This is the first study reporting a knockout of the intestinal epithelial cell-specific *Piga* gene (*Piga-/-*) and its association with the gut microbiota in mice using a whole metagenome shotgun-based sequencing approach*.* An overall reduced microbiota diversity has been observed in the *Piga-/-* group as compared to the control group (ANOVA *p* = 0.34). The taxonomic biomarkers, namely: Gammaproteobacteria (class), Enterobacterales (order), *Enterobacteriaceae* (family), *Escherichia* (genus), *Proteus* (genus) and *Escherichia coli* (species), increased more in the *Piga-/-* mice as compared to in the control group. Further, the pathogenic *E. coli* strains, namely *E. coli* O157:H7 str. EDL 933 (EHEC), *E. coli* CFT073 (UPEC) and *E. coli* 536 (UPEC), were found in the *Piga-/-* mice which also harbored virulence factor transporters. In addition, the taxa responsible for short chain fatty acid production were decreased in the *Piga-/-* group. The *Piga-/-* mice gut harbored an increased number of microbial functions responsible for the survival of pathogens in the inflamed gut environment. Our observations clearly indicate that the *Piga-/-* mice gut might have an overall enhancement in pathogenic behaviour and reduced capabilities beneficial to health.

## Introduction

The microbes and their cumulative genomes, called the microbiome, modulate metabolic phenotype and influence the host immune system^1^. The interactions between gut microbiota and the immune system commence at birth. The gut micobiota influence the development of the immune system; and the immune system in turn shapes the gut microbiota composition^2^. Commensal bacteria act on the host's immune system to induce protective responses that prevent colonization and invasion by pathogens^3^. In genetically susceptible individuals atypical interactions between the microbiome and the host’s immune system may contribute to the development of complex immune-mediated diseases, including inflammatory bowel disease (IBD), systemic autoimmune diseases, and cancer, etc.^4^. For example, the dysregulated microbiome-immunity interaction associated with *NOD2* mutation is assumed to play an important role in Crohn’s disease pathogenesis^4^.

Glycosylphosphatidylinositol (GPI) is a glycolipid, which is produced in the endoplasmic reticulum (ER) and to the proteins^5^. The GPI-anchors are involved in important processes, including protein trafficking, sorting, and dynamics. A large number of GPI-anchored proteins (GPI-APs) have been identified in higher eukaryotes including human, *Arabidopsis thaliana*, mouse, and rat. The GPI-APs function as enzymes and complement regulators, adhesion molecules, and co-receptors in the signal transduction pathways^6^. These proteins play pivotal roles in several physiological processes such as embryogenesis, neurogenesis, fertilization, and immune responses^7–11^.

With respect to the intestinal immune response, some GPI-APs are known to activate important immune molecules and play pivotal roles in host gut health. On the other hand, there are some GPI-APs which are known to promote the growth of commensal microbiota^12^. In addition to these, the GPI anchor has also been found as one of the dominant sorting signals in intestinal epithelial cells (IECs)^13^ and GPI-APs may partially contribute to IECs polarity^14^. The polar nature of IECs is essential for the establishment of the epithelial barrier function, which is required to segregate the commensal microbiota from the host’s internal milieu^14^. Some of the GPI-APs, including glycoprotein 2 (GP2) and Ly6/PLAUR domain containing protein 8 (Lypd 8) are reported to be involved in microbial defense and play important roles in the intestinal immune system and homeostasis^15,16^.

For the biosynthesis of GPI-APs, eukaryotes have conserved post-translational mechanism, which is critical for attaching these proteins to the cell membrane^5^. Over 20 phosphatidylinositol glycan biosynthesis protein (PIG) subclasses involved in the biosynthesis and remodeling of GPI-anchors have been discovered so far^5^. The first step of GPI-anchor biogenesis is the formation of N-acetylglucosamine-phosphatidylinositol with the help of an enzyme which is encoded by a cluster of seven genes including the *PIGA* (MIM 311,770) gene^5^. Mutations in the GPI biosynthesis genes have been linked to a growing number of human diseases. For example, somatic and germline mutations in the *PIGA* gene have led to GPI deficiencies^6,17^ in PNH (paroxysmal nocturnal hemoglobinuria)^17^ and XLIDD (X-linked intellectual developmental disorder)^6^, respectively.

Given such important roles of the GPI-APs and gut microbiome in intestinal immune homeostasis, an exploration of the effect of one on the other will help in deciphering the possible cross-talks between these two important contributors to host health. To this end, for the first time, we developed an intestinal epithelium-specific *Piga* gene knockout in mice since this gene is known to play an important role in GPI-anchor biogenesis process. We hypothesize that by knocking out the *Piga* gene, the expression and localisation of the intestine-specific GPI-APs may be hampered, leading to a compromise in the intestinal immune system which may be associated with gut microbiota dysbiosis. The main aim of the present study is to perform a comparative analysis of the gut microbiota of the *Piga* gene knockout mice with the control mice to explore the microbial community dynamics and the respective microbial functional alternations and to gain important insights on the possible cross-talk between the GPI-APs and mice gut microbiota. To address this, we have used the whole metagenome shotgun sequencing (WMGS) based approach to perform the metagenomic sequencing of the gut microbiomes of *Piga-/-* and control mice. The scope of this analysis is to perform a comprehensive comparative metagenomic analysis of the gut microbiota of the two groups to elucidate the important microbial changes and their functional dynamics which may be associated with the impact of the knockout of the *Piga* gene on intestinal immune homeostasis. Such analysis will help us in the identification of the possible cross-talk between the two important contributors of intestinal immunity namely, the gut microbiota and GPI-APs.

## Results

### Taxonomic identification of metagenomic samples in control and *Piga-/-* mice

We investigated the effect of *Piga* gene knockout on the composition of fecal microbiota using whole metagenome shotgun sequencing (WMGS) analysis at all taxonomical levels (S1 Fig. and S1 Table). One of the major advantages of the WMGS approach lies in the availability of functional information and taxonomic data for the microbiome. The sequenced reads were processed to remove low quality reads, duplicated reads, and contaminations of host DNA. The remaining good quality metagenomic reads were mapped on bacterial genomic data to obtain taxonomic and functional feature counts. The obtained feature counts (taxonomic and functional) were normalized and statistical analyses were performed. A total of 2.29 billion raw reads were obtained as a result of metagenomic sequencing which resulted in 1.54 billion good quality reads. Based on the alpha-diversity analysis, the diversity of the *Piga-/-* samples is found to be lower than the controls, albeit with a p-value greater than the significance cut-off (p = 0.34 ANOVA S2 Fig.).

**Figure 1:**
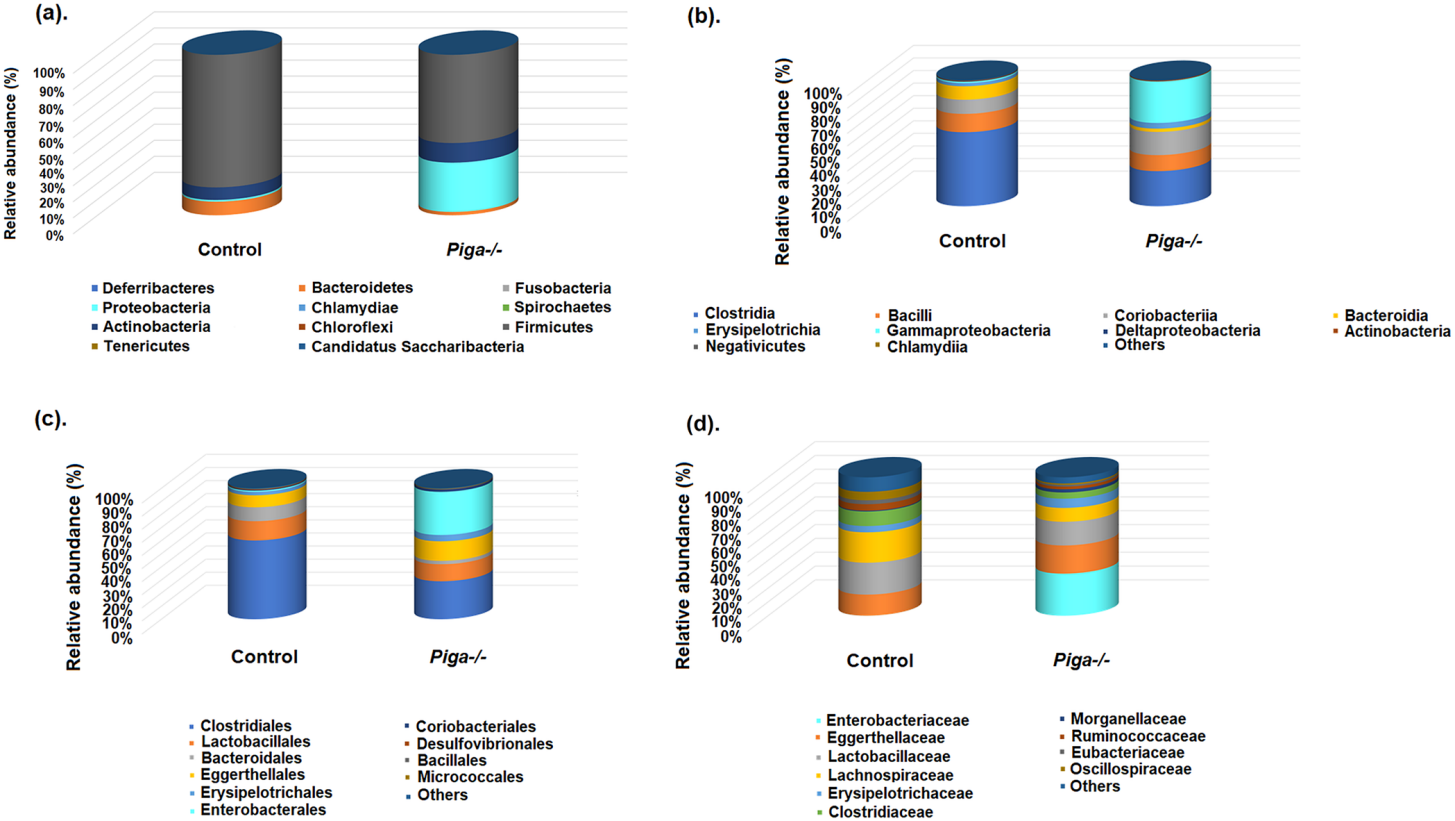
Relative abundances of taxa at the (**A**) phylum, (**B**) class, (**C**) order, and (**D**) family levels in the *Piga-/-* and control metagenomic samples.

Metagenomic analysis of the samples showed that the relative abundance of phyla Proteobacteria (*Piga-/-*—> 30.67%; control—> 1.01%) and Actinobacteria (*Piga-/-*—> 12.22%; control – > 7.78%) increased whereas that of Firmicutes (*Piga-/-* – > 54.93%; control – > 82.59%) and Bacteroides (*Piga-/-* – > 2.07%; control – > 8.45%) decreased in the *Piga-/-* samples as compared to the control group (Fig. 1). We also explored which taxa were predominantly altered at the class, order, and family levels using the taxa relative abundances (Fig. 1 and S2 Table). An F-test analysis indicated significant alterations in the relative abundance of the order Enterobacterales (*p*-value 0.001561) and family *Enterobacteriaceae* (p-value 3.87995 E-06) (S2 Table). At the other levels alterations in the taxa abundances are clearly observed although no statistical significance was obtained using the F-test.

As shown in Fig. 1, the relative abundances of family *Lactobacillaceae, Clostridiaceae,* and *Lachnospiraceae* belonging to phylum Firmicutes and order Bacteroidales belonging to phylum Bacteroidetes are higher in the control group than in the *Piga-/-* samples. This corroborates earlier observations that a healthy gut is dominated by Firmicutes and Bacteroides taxa in mice^18^. On the other hand, *Enterobacteriaceae, Eggerthellaceae,* and *Erysipelotrichaceae* of phyla Proteobacteria, Actinobacteria, and Firmicutes, respectively, are increased in the *Piga-/-* group more than in the control metagenomic samples. An increased abundance of taxa Actinobacteria and Proteobacteria correlates with a diseased gut as in the case of IBD^19^. However, an increased abundance of *Erysipelotrichaceae* of phylum Firmicutes is noteworthy. *Erysipelotrichaceae* have been found to be enriched in colorectal cancer and changes in their abundance have also been identified in patients with IBD or animal models of IBD^20^. These studies emphasize the importance of *Erysipelotrichaceae* in inflammation-related gastrointestinal disorders.

**Figure 2:**
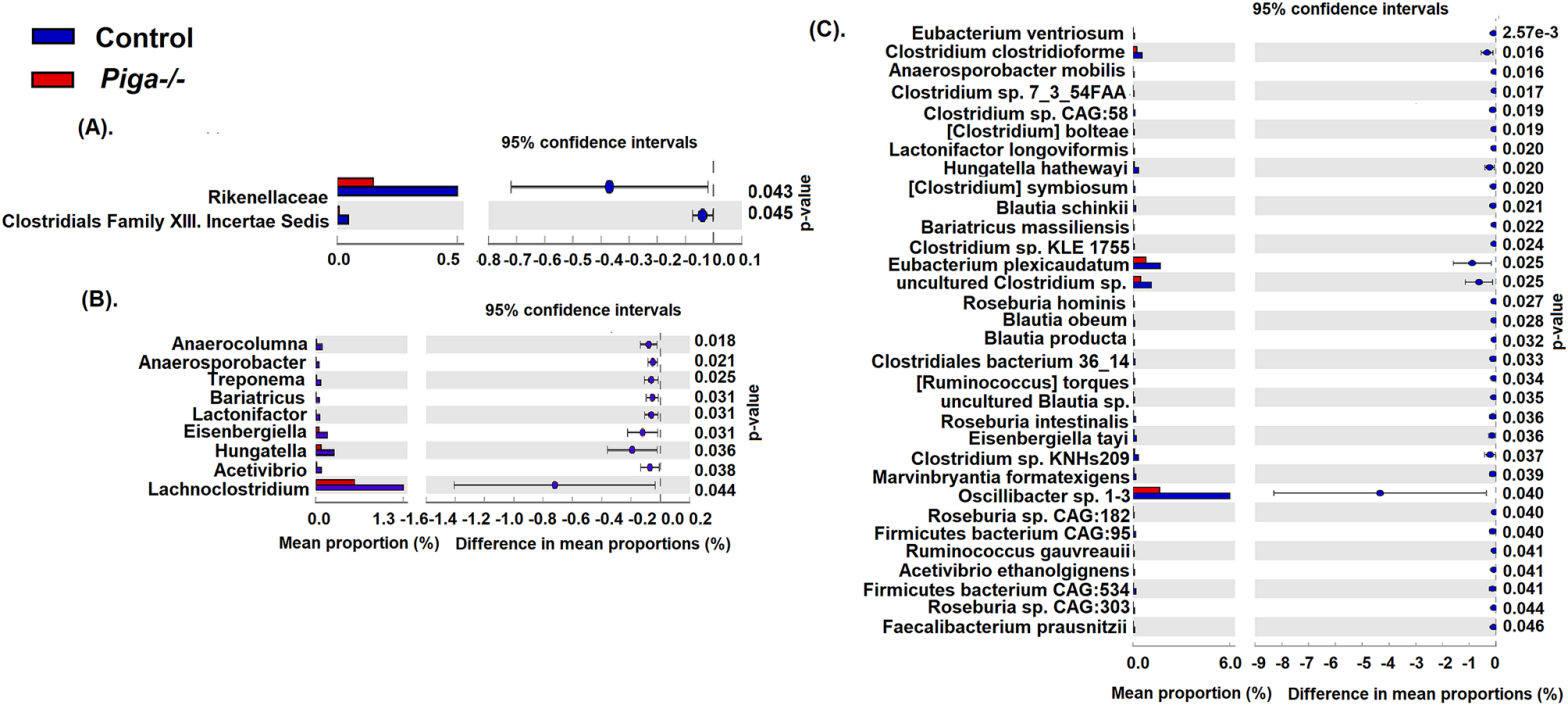
Extended bar chart represents statistically differing taxa at (**A**) family, (**B**) genus, and (**C**) species levels. Welch’s t-test was performed with *p* < 0.05 and a confidence interval of 95%.

We used another method, Welch’s t-test on the differences in mean-proportions of taxa abundance to examine significant differences in the microbiota composition at various taxonomic levels between the *Piga-/-* and control metagenomic samples (Fig. 2). Using this method, at the phylum, class, and order levels no taxa were found to be significantly altered statistically. At the family, genus, and species level all the significantly differing taxa were found to be decreasing in the *Piga-/-* as compared to the control metagenomic samples (Fig. 2A–C). Most of the altered taxa belong to the *Lachnospiraceae, Ruminococcaceae, Clostridiaceae,* and *Eubacteriaceae* families (S3 Table). Some members of these families have been noted for their active participation in the production of short chain fatty acids (SCFAs), specifically butyrate^21^. The remaining non-butyrogenic taxa may influence butyrate production by making other butyrate precursors such as acetate, lactate, and succinate (S3 Table) while some others may be involved in propionate production^22^. This indicates that in the *Piga-/-* mice the population of beneficial gut commensal microbes is reduced and subsequently their potential for butyrate production in addition to the other SCFAs also is diminished.

**Figure 3:**
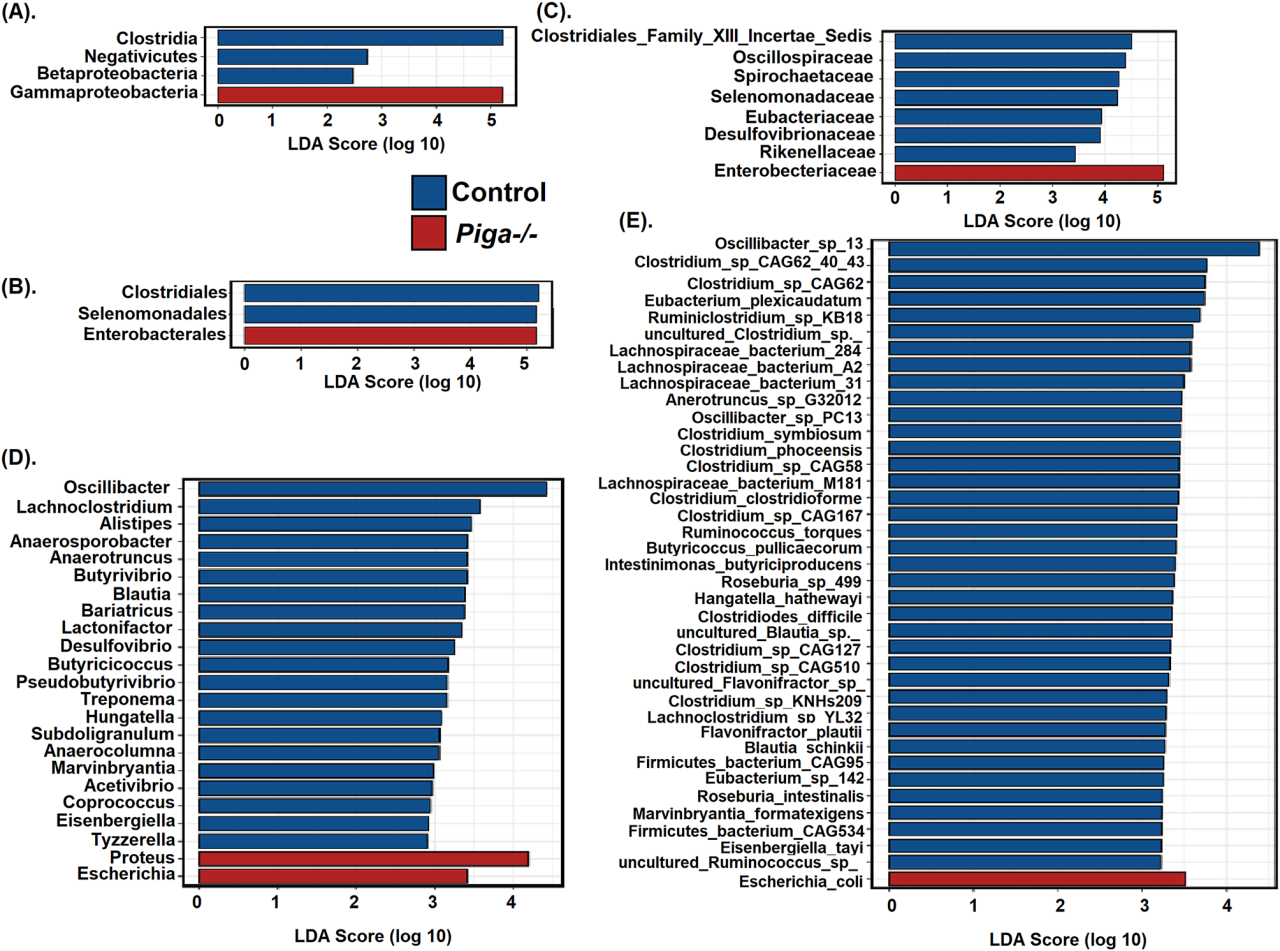
Differentiating biomarkers identified using the LEfSe analysis on different levels of taxonomy, namely, (**A**) class, (**B**) order, (**C**) family, (**D**) genus, and (**E**) species.

To determine the differentiating biomarker taxa specific to the *Piga-/-* and control groups a LEfSe analysis was performed on the metagenomic reads (Fig. 3). This analysis identified taxonomic biomarkers at all levels, except at the phylum level. At the species level only one taxa, namely *Escherichia coli*, was found to be a biomarker specific to the *Piga-/-* group. Consistent with this observation, *Escherichia* was identified as one of the biomarkers at the genus level in the *Piga-/-* group as well. Further, *Enterobacteriaceae,* Enterobacterales*,* and Gammaproteobacteria were identified as the biomarkers at the family, order, and class levels, respectively, in the *Piga-/-* group. In addition to *Escherichia*, *Proteus* were also identified as the biomarker for the *Piga-/-* group at the genus level. These observations clearly demonstrate that the gram-negative bacterial population is profoundly increased in the *Piga-/-* mice as compared to the control. Interestingly, some of the members of genus *Escherichia*, and *Proteus* are known to be potential pathogens in gut associated diseases. For example, *E. coli* and *Proteus spp.* have been suggested to play a role in the pathogenesis of IBD^23^. This indicates that in the absence of the *Piga* gene the epithelial barrier breach by pathogenic microbes might be increased, leading to a rise in their population in the *Piga-/-* mice.

### Functional identification in Control and *Piga-/-* metagenomic samples

The functional prediction for the metagenomic reads was performed by mapping the reads on the EggNOG functional classes. In order to explore the microbial functional variations in the *Piga-/-* as compared to the control samples, Welch’s t-test was performed on the predicted functions based on the EggNOG classification of the metagenomic reads. The statistically significantly different functions at the levels -1 and -2 were not identified (S3 Fig.). Using Welch’s t-test analysis, at the level -3, we found 226 significantly altered COGs/NOGs belonging to cellular processes and signaling, information storage and processing, and metabolism functional classes (S4 Table, S4 Fig.).

In this analysis, at level -3 a large majority of the COGs/NOGs were found to be decreasing in the *Piga-/-* as compared to the control samples. Among these are the genes involved in cobalamin (vitamin B12) biosynthesis (COG2243, COG1492, COG2875, COG1270, COG1797, COG2242, COG2082, COG2038), biotin metabolism (COG1654, ENOG410XQH3), riboflavin metabolism (COG0807), nicotinate and nicotinamide metabolism (COG1712) and propanoate metabolism (COG1803, COG0511). This suggests that the gut microbiota associated with the *Piga-/-* mice have lost their capability to produce cofactor and SCFAs as compared to the control mice. In addition, COGs involved in other processes such as defense mechanisms (ENOG410ZX9X, ENOG410XTHI, ENOG410ZYVS, ENOG410ZVEA, ENOG4111FSE, ENOG410XQ2T, ENOG410Y2F5, ENOG410Y12H, ENOG41120ZG, ENOG410ZWUI, ENOG410YATZ, ENOG410XTDY, ENOG410ZWZY), intracellular trafficking, secretion, and vesicular transport (ENOG41122HV, ENOG410XR7W, ENOG41124QY, COG3505) and signal transduction mechanisms (ENOG4111VXS, ENOG4111HM8, COG0643, COG4252, ENOG4111GNT, COG2172, ENOG4111Y53, ENOG410XPTB, ENOG410YSNH, ENOG410ZWIB, COG5001, ENOG41120GI) are also found to be significantly reduced in the *Piga-/-* as compared to the control samples.

Only 11 COGs/NOGs were found to be significantly increased in the *Piga-/-* as compared to the control metagenomic samples (S4 Table). Ribose-5-phosphate isomerase A (RpiA: COG0120) (S4 Table) is found to be significantly increased in the *Piga-/-* mice gut as compared to the control. This enzyme interconverts ribose-5-phosphate and ribulose-5-phosphate and plays critical roles in the anabolism and catabolism of carbohydrates (especially in the pentose-phosphate pathway). Pentose-phosphates are the crucial intermediates of the cellular-metabolism of *E. coli*^24^. The production of the heptose components of lipopolysaccharide (LPS), as well as other essential components, requires RpiA activity^24^. This indicates an enhanced growth of LPS covered bacteria in the knockout mice gut. The other functions that may aid bacterial survival in an inflamed gut are also found to be significantly increased in the *Piga-/-* mice gut as compared to the control (Table 1). It is interesting to note that the taxonomic biomarkers in the *Piga-/-* mice were gram-negative bacterial populations. The LPS covering of these gram-negative bacteria might be responsible for the inflammation in the gut and subsequently these bacteria might produce the observed genes that will allow them to reside in the inflamed gut. These findings indicate that in the gut of knockout mice, where the immune system was already weakened, the microbiota lowered the expression of genes implicated in processes like defense, signaling and transport and trafficking and might utilize their energy in enhancing those genes that aid in their overall survival in the inflamed gut.

**Table 1:** Significantly increased microbial functions in the *Piga-/-* mice gut microbiota that might help these bacteria in their survival in an inflamed gut.

COGs	Roles
Fumarate hydratase class II (COG0114)	Involved in the TCA cycle and its expression was observed in conditions of iron limitation and oxidative stress^25^
YhcH YjgK YiaL family protein (COG2731)	YjgK (TabA) influences biofilm formation by toxin-antitoxin systems^26^. YhcH is a possible sugar isomerase of sialic acid catabolism^27^
Part of the ABC transporter complex PotABCD involved in spermidine putrescine import (COG3842)	Spermidine accumulation increases resistance to oxidative stress in gram-negative bacteria and their survival in macrophages^28^
Polypeptide-transport-associated domain protein, ShlB-type (COG2831)	ShlB is important in the secretion and activation of the haemolysin ShlA (cell bound hemolysin, releases heme–iron from erythrocytes by interaction with erythrocyte membrane)^29^

### Backtracing of significantly altered functions on significantly altered taxa

Aiming to understand which taxa may be primarily responsible for the alternations in microbial functions observed in the *Piga-/-* mice gut microbiota as compared to the control, we performed a backtracing analysis. In addition, the backtracing analysis helps in identifying the microbial strain level differences. In this analysis, we explored which significantly altered bacterial taxa harbored the significantly altered functions observed in the *Piga-/-* mice gut microbiota. Out of the 215 COGs/NOGs, which are found to be significantly increased in the control metagenomic samples, 142 functions backtraced on 12 taxa belonging to eight distinct bacterial species (S5 Table). These species include *Eubacterium ventriosum* ATCC 27,560, *Clostridium bolteae* ATCC BAA-613, *Clostridium symbiosum* WAL-14163, *Marvinbryantia formatexigens* DSM 14469, *Roseburia hominis* A2-183, *Roseburia intestinalis* XB6B4, *Ruminococcus torques* ATCC 27756, *Ruminococcus torques* L2-14, *Faecalibacterium prausnitzii* L2-6, *Faecalibacterium cf. prausnitzii* KLE1255, *Faecalibacterium prausnitzii* SL3/3, and *Faecalibacterium prausnitzii* A2-165. As it is evident from S3 Table that several of these bacterial species are actively involved in SCFA production.

It is interesting to note that all the 11 significantly altered COGs/NOGs, which are found to be increased in the *Piga-/-* metagenomic samples, backtraced on different strains of the *E. coli* (S6 Table). It is noteworthy that *E. coli* is found as the taxonomic biomarker for the *Piga-/-* group as compared to the control in our analysis. This clearly indicates that *E. coli* is one of the prime taxa which is increased in the *Piga-/-* mice and might start to express those functions which will help it to survive in the inflamed gut. The strains of *E. coli* which are identified in this analysis include *E. coli* O157:H7 str. EDL 933 (EHEC), *E. coli* CFT073 (UPEC), *E. coli* 536 (UPEC), *E. coli* str. K-12 substr. DH10B, *E. coli* str. K-12 substr. W3110, *E. coli* str. K-12 substr. MG1655, *E. coli* ATCC 8739, and *E. coli* BL21 (DE3). Among the above *E. coli* strains, *E. coli* O157:H7 str. EDL 933 (EHEC), *E. coli* CFT073 (UPEC), and *E. coli* 536 (UPEC) are the pathogenic strains^30^. In addition, these are the only strains which mapped on the polypeptide-transport-associated domain protein, ShlB-type (COG2831) function, which is a virulence factor transporter. This clearly indicates that in the absence of the *Piga* gene these three *E. coli* strains might be the prime pathogenic strains which might breach the epithelial barrier and further contribute to gut inflammation.

## Discussion

### Role of GPI-APs in gut-associated immune processes

In order to understand the cross-talk between the gut microbiota and intestine specific GPI-APs, we have created intestinal epithelium-specific *Piga-/-* mice and explored the microbiota dynamics with respect to the control in the present analysis. Several members of the GPI-APs family are expressed on the apical or basolateral surfaces of the IECs. GPI-anchors play an important role as a signal for the transport of proteins to the apical surface of the fully polarized epithelial cells^14^. A loss of the *Piga* gene due to knockout is expected to result in an impaired GPI-anchoring process. This in turn might lead to diseases by affecting the expression and localisation of various GPI-APs in the intestine and the important functions imparted by them. For example, the mislocalisation of the apical proteins in IECs leads to malnutrition and diarroheal disroders^31^. On the other hand, the mislocalisation of basolateral proteins correlates with loss of epithelial architecture, cancer development, and IBD. Although not confirmed, the mislocalisation of α2-integrin, which is a basolateral GPI-AP, has been reported in some microvillus inclusion disease (MVID) patients and mouse models.

The GPI-APs are known to play several important roles in overall cell biology, including intestinal immunity (S7 Table). IECs are constantly challenged by a bombardment of foreign antigens and environmental microorganisms. The epithelial barrier not only carries out pathogenic microbial surveillance and prevents their breach from the lumen across the barrier, but also maintains immune tolerance to commensal microbes. A precise regulation of the intestinal epithelial barrier allows the maintenance of mucosal immune homeostasis and prevents the onset of uncontrolled inflammation. The functions of epithelial cells are executed by their polar nature, which are generated in part by GPI-APs^14^. A loss of epithelial cell polarity is associated with impaired epithelial homeostasis and has been linked with several diseases^32,33^. Similarly, a defective microbial surveillance mechanism in the intestinal lumen is known to result in a loss of mucosal immune homeostasis leading to an inflamed gut^33^. Those GPI-APs which are present on the apical surface come in contact with the gut microbiota or the other microbes that enter into our bodies through our digestive tracts. In this context, glycoprotein 2 (GP2), Ly6/PLAUR domain-containing protein 8 (Lypd8), uromodulin (UMOD), cellular prion protein (PrP^C^), and β1 Integrin are particularly interesting GPI-APs since they are all present on the apical surface of the M-cells of the epithelial layer (S7 Table).

Lypd8 is a GPI-AP which is a secretory protein specifically needed to prevent the invasion of gram-negative flagellated bacteria in the inner-mucus layer. It binds to the flagellum of bacteria, such as *Helicobactor*, *Escherichia,* and *Proteus* (*P. mirabilis*), thereby inhibiting bacterial motility in the intestinal lumen. Segregation of epithelial cells and intestinal bacteria is required to keep intestinal homeostasis in the colon^16^. *Lypd8 − / − *mice were found to be extremely sensitive to dextran sulfate sodium (DSS) induced intestinal inflammation. Antibiotic administration eliminated the gram-negative flagellated bacteria, re-established the bacterial free state of the inner-mucus layer, and ameliorated intestinal inflammation induced by DSS in these mice. Recently, Lypd8 deficiency has been shown to promote the rapid colonization of *Citrobacter rodentium* (family *Enterobacteriaceae*) in the colon resulting in severe colitis with neutrophil and Th17-cell expansion in the lamina propria^34^. Lypd8 prevents *C. rodentium* from attaching to the intestinal colonic epithelial cells, thereby preventing intimin and translocated intimin receptor interaction^34^. Hence, Lypd8 plays an important role in early-phase defense against *C. rodentium*^34^.

The GP2 protein is a GPI-AP which is expressed on the M cells and acts as a bacterial uptake receptor. A previous study has demonstrated that it mediates antigen transcytosis by M cells in the mucosal-associated lymphoid tissue^15^. It specifically recognizes FimH, on the outer membrane of a subset of gram-negative enterobacilli such as *E. coli* and *Salmonella enterica*^15,35^. However, recently it has been found that the Pancreatic acinar cells and not M cells, are the source of intestinal GP2^36^. Pancreas-specific GP2-deficient colitis mice have a larger mucosal *E. coli* population than the intact mice, indicating that the digestive-tract GP2 binds the commensal *E. coli*, preventing the epithelial attachment and penetration by this pathogen. However, both the pancreas-specific and intestinal epithelium-specific GP2-deficient mice do not exhibit any significant alterations in the luminal microbiota content as compared to the wild type mice. Thus, although the intestinal epithelium M-cell specific GP2 have been shown to be involved in FimH + bacterial recognition and their subsequent translocation, their roles in the mechanism of microbial surveillance and clearance remains poorly understood and requires further exploration.

UMOD is another GPI-AP, which is closely related to GP2 and shows binding towards the FimH + Type 1 pili. This gene was first characterized as the Tamm-Horsfall protein expressed on the apical surface of renal tubular epithelial cells and capable of being shed into urine where it binds to uropathogenic *E. coli*, interfering with bacterial adhesion and pathogenicity^37,38^. However, the M-cells specific UMOD serve as an uptake receptor for *Lactobacillus acidophilus* (L-92 strain), a probiotic strain known to exert anti-allergic immunomodulatory effects upon oral administration in mice^39^. The interaction of the surface layer protein A (SlpA) on L-92 with UMOD on the M-cell surface is responsible for M-cell mediated uptake of L-92 and the bacteria is subsequently delivered to the Peyer’s patches (PP) dendritic cells for immunomodulation. These observations suggest that bacterial access to the gut immune system is a crucial process to promote host immune responses and some GPI-APs play crucial roles in such processes. Corroborating with this role of UMOD, in our study, we have noted an overall reduced relative abundance of *Lactobacillus* genera in the *Piga-/-* mice group.

Although the proteins mentioned above serve a crucial function in microbial surveillance or immunomodulation processes, some GPI-APs have been found to be exploited by pathogenic bacteria. *Brucella abortus* is a Gram-negative bacterium causing a form of brucellosis in cattle and other domestic animals which results in their transmission to humans. PrP^C^ is a GPI-AP which is expressed on M-cells and acts as an uptake receptor for *Brucella abortus* (via binding with the conserved Hsp60 proteins expressed on *B. abortus*)^40^. Following *B. abortus* uptake, the bacteria penetrate into M cells and are capable of surviving inside Dendritic Cells (accumulated beneath the M cells) by producing replicative vacuoles which then spread throughout the body. Similarly, the GPI-linked protein integrin β1, which is normally distributed in the basolateral membrane of enterocytes, localizes in the apical surface of M cells, and acts as a receptor for the pathogenic *Yersinia enterocolitica*^38^.

There are a number of intestine specific GPI-APs whose membrane location is not known. These include T-cell ecto-ADP-ribosyltransferase 1, T-cell ecto-ADP-ribosyltransferase 2, and low affinity immunoglobulin gamma Fc region receptor IV which are known to activate the important immune molecules such as macrophages and T-cells (S7 Table). GPI-APs including, hydrolases and proteases and their receptors, play important roles in host gut health (S7 Table). It is interesting to note that some of the basolateral proteins such as TLR5 and TLR9 can act as pattern recognition receptors for microbial pathogens^41^. Thus, the roles of the above-mentioned GPI-APs in microbial recognition and other similar processes cannot be ruled out and requires further exploration.

One of the GPI-APs, namely intestinal-type alkaline phosphatase (IAP), is found to promote the growth of commensal microbiota and maintain overall gut immunity. IAP is a small intestinal brush border enzyme which has been recognised as a gut mucosal defense factor. IAP has the ability to detoxify lipopolysaccharides (LPS) from Gram-negative bacteria and exogenous IAP has been shown to attenuate LPS-mediated toxicity^42,43^. Further, an IAP knockout (IAP-KO) mice study reported increased bacterial translocation to mesenteric lymph nodes when the intestine was subjected to a local or distant ischaemic injury^44^. The luminal environment of IAP-KO mice is unfavorable particularly for common enteric commensal *E.* coli^44^. Also the gut of IAP-KO mice have more Clostridia belonging to the Firmicutes phylum than the wild type^44^.

GPI-AP Intelectin have been implicated in innate immunity and are mainly produced by intestinal goblet cells, intestinal Paneth cells, and lungs^45–47^. In mice, expression of intelectin rises upon intestinal parasitic nematode infection^48^. Intelectins are also found abundantly in the mucus produced by allergic responses in humans^49^. Recently, human intelectins (hIntL-1) are found to differentiate between the glycans found on mammalian cells from those of microorganisms resulting in the recognition of the later^50,51^. The ligands of hIntL-1 include glycans from Gram-positive and Gram-negative bacteria, including *Streptococcus pneumoniae*, *Proteus vulgaris*, *Proteus mirabilis*, *K. pneumoniae*, and *Yersinia pestis*^50,51^. This selectivity of only microbial ligands suggests that hIntL-1 functions in the microbial surveillance process. Further, mouse IntL-1 is reported to interact with the β-Galf gene^50,51^. These findings support the hypothesis that IntLs from diverse organisms have evolved to bind specific microbial epitopes and may play defensive roles against microbes.

The above discussion suggests that several intestine specific GPI-APs have important roles to play in intestinal immune homeostasis. Some GPI-APs which are present on the basolateral membrane or plasma membranes of the intestine may be involved in gut immunity either by directly or indirectly associating with the microbial members. Also, several GPI-APs which are present on the apical surface play a variety of important roles in maintaining gut- associated immunity including microbial surveillance by acting as pathogen uptake receptors or immunomodulation processes. On the other hand, some apical GPI-APs are utilized by pathogens for their uptake into the host system. Therefore, the dysregulation of the expression and localisation of these GPI-APs due to the *Piga* knockout is expected to severely affect all these immune related process and affect the overall host health.

### Family *Enterobacteriaceae* and species *E. coli* as the differentiating biomarkers of the *Pig-/-* mice from the control

The above discussions suggest that an impaired function of GPI-APs including, Lypd8, UMOD, and perhaps GP2 and others is expected to lead to a faulty pathogenic microbial surveillance. Since these proteins are involved in defense against pathogenic bacteria such as *E. coli*, *Proteus*, *H. pylori*, and *S. enterica* by various mechanisms, a loss in the functioning of these proteins will lead to an increased abundance of these pathogens. This is mainly because these pathogens might escape from the recognition by the immune molecules which would have otherwise eliminated them. We have found the family *Enterobacteriaceae* and species *E. coli* as the taxonomic biomarkers in the *Piga-/-* mice group. Another pathogenic genera, namely, *Proteus* is also identified as a biomarker with the relative abundance of the species *Proteus mirabilis* increased in the *Piga-/-* mice. Further, in the *Piga-/-* mice microbial functions that help in the survival of pathogenic microbes in an inflamed gut are found to be significantly increased. In addition, we have observed an increased relative abundance of the fimbrial proteins (COG3539, COG4969) in the *Piga-/-* mice.

Interestingly, the functions increased in the *Piga-/-* mice in our backtracing analysis are found to be mapping on only *E. coli* species including the three pathogens namely, *E. coli* O157:H7 str. EDL 933 (EHEC), *E. coli* CFT073 (UPEC), and *E. coli* 536 (UPEC). All these pathogenic *E. coli* strains are found to harbor a virulence factor transporter. A recent study showed that Enteropathogenic *E. coli* (EPEC) can disrupt cell polarity, causing basolateral membrane proteins, in particular β_1_-integrins, to migrate to the apical cell surface. These β_1_-integrins on the apical cell surface can bind to the intimin of EPEC, which mediates the intimate attachment of EPEC to epithelial cells^52^. Although Uropathogenic *E. coli* (UPEC) is responsible for causing urinary tract infection, it is likely that the infection begins with the colonization of the bowel with a uropathogenic strain in addition to the commensal flora.

From these discussions, we conclude that due to the *Piga* gene knockout the pathogenic bacteria will escape the host immune system by preventing their recognition and transportation through the immune molecules (for example, due to the lack of Lypd8 protein) and subsequently this will lead to a gut microbiota dysbiosis. Since the GPI-APs such as GP2, which are actively involved in the surveillance of pathogenic *E. coli* in gut are of pancreatic origin, a knockout of intestinal *Piga* is not expected to impact the function of the GP2 of pancreatic origin. However, we observe a significantly higher abundance of pathogenic *E. coli* in *Piga-/-* mice group. This clearly indicates that the process of microbial surveillance is performed by other factors (Lypd8 and UMOD and others) than the pancreatic GP2 proteins. As the abundance of pathogenic *E. coli* species increases in the gut, strains such as EPEC can further disrupt the epithelial cell polarity, thereby aggravating the condition further. This also indicates that gut inflammation may be one of the phenotypes associated with the intestine specific *Piga-/-* in mice, mainly due to the increase in the abundance of pathogenic microbes.

### *Piga-/-* mice gut microbiome exhibits a reduced ability for SCFA production

Another interesting observation is a significantly reduced abundance of the butyrate and propionate producing commensal bacteria in the *Piga-/-* mice group. These include *F. prausnitzii, R. intestinalis, R. hominis, E. ventriosum, E. plexicaudatum, R. torques, B. massiliensis, E. tayi, C. symbiosum, C. clostridioforme, Clostridium sp. KNHs209* and *Hungatella hathewayi.* Further, the significantly increased microbial functions in the control mice backtraced predominantly on those commensal species which were responsible for SCFA production. These observations suggest that in the *Piga-/-* mice the population of beneficial gut commensal microbes may get reduced and subsequently their potential for butyrate and other SCFAs production may also get diminished. This suggests that the intestinal mucosa of the *Piga-/-* mice have lost their energy source that could have been provided by these commensal bacteria. Butyrate is a critical metabolite for the overall gut physiology and host wellbeing. It is the main source of energy for the colonic epithelial cells, and it has defensive properties against IBD and colorectal cancer (CRC). Butyrate can reduce mucosal inflammation through upregulating peroxisome proliferator-activated receptor (PPAR)-gamma, inhibiting NF-κB transcription factor activation, and interferon gamma^53^. In addition, propionate has been demonstrated to be converted into glucose through intestinal gluconeogenesis (IGN) thus further improving the energy homeostasis^54^.

*F. prausnitzii* is a representative commensal member of the phylum Firmicutes and is well-known for playing a number of important functions in gut homeostasis. In a previous study the administration of *F. prausnitzii* (strain A2-165) or its supernatant significantly reduced colitis severity induced by trinitrobenzenesulfonic acid (TNBS) and restored dysbiotic gut microbiota community^55^. In addition, *F. prausnitzii* (strain A2-165) exerted anti-inflammatory effects by inhibiting NF-κB activation and IL-8 production (on both cellular and colitis animal models)^55^, facilitating the IL-10 induction in murine and human dendritic cells and altering T-cell responses^56^. Furthermore, in colitis mice models, *F. prausnitzii* (strain A2-165) or its supernatant has been shown to reduce colitis severity by downregulating pro-inflammatory cytokines, myeloperoxidases, and T-cell levels, as well as maintaining the intestinal epithelial barrier^57,58^. *F. prausnitzii* may be a promising candidate for next-generation probiotics because of these characteristics^59^. In corroboration with these observations, we found significant reduction in *F. prausnitzii* in our *Piga-/-* group, leading us to suspect that *Piga-/-* mice gut might have lost the probiotic community of microbes and their subsequent beneficial anti-inflammatory effects.

*Roseburia spp*., along with *F. prausnitzii* and *Eubacterium spp.*, form a prominent butyrate-producing Firmicutes group that accounts for 7–24% of all bacteria in a healthy human colon^60^. A previous study reported that the intestinal-abundance of *R. intestinalis* was much lower in untreated CD patients than in healthy controls^61^. To reduce intestinal inflammation, *R. intestinalis* raised the number of T-regulatory (Treg)-cells and enhanced the gene expression of the cytokines, namely, transforming growth factor-β (TGF-β) and thymic stromal lymphopoietin (TSLP)^61^. In addition, *R. intestinalis* supernatant was observed to ameliorate colitis in TNBS and DSS-induced mice models by decreasing the count of Th17 cells and inflammatory macrophages in the colon, and by suppressing the gene expression of signal transducer and activator of transcription 3 (STAT3) and IL-6^62^. In addition, *R. hominis* (strain A2-183) promotes and regulates innate immunity via upregulating genes related to gut barrier function, antimicrobial peptides, T-cell biology, and toll-like receptors (TLR) signaling^63^. Furthermore, treatment with *R. hominis* provided protection against DSS-induced colitis^63^. Additionally, patent rights have been granted for the use of several strains of *E. ventriosum* in the treatment of colitis and/or colorectal cancer^64^. In our study we found a significant reduction in *Roseburia spp*., and *Eubacterium spp.* in the *Piga-/-* group as compared to controls leading us to hypothesize that the commensal community of microbes and their subsequent beneficial anti-inflammatory and immunomodulatory effects are lost in *Piga-/-* mice gut.

On the other hand, we found *E. coli* O157:H7 str. EDL 933 (EHEC), *E. coli* CFT073 (UPEC) and *E. coli* 536 (UPEC) pathogenic strains as a taxonomic biomarker in *Piga-/-* group. These observations corroborate the fact that colonic SCFA regulate the growth and virulence of enteric pathogens, such as enterohemorrhagic *E. coli* (EHEC)^65^. A reduced ability to produce SCFAs has been shown to be linked with gut microbiota dysbiosis and an increased abundance of microbes with deleterious effects on host health^66^.

This is the first study of the intestinal epithelium specific *Piga* gene knockout creation in mice and its impact on the fecal microbiota by using the whole metagenome shotgun sequencing method. In *Piga-/-* mice, we were able to identify the main features of microbiota dysbiosis in terms of taxa and gene functions. The findings in this paper provide new directions for designing more focused experiments to confirm the microbial and functional changes associated with the *Piga-/-* and gut health.

## Materials and methods

### Animal experiments, knockout preparation, and sample collection

All animal experiments were approved by the Institutional Animal Care and Use Committee (IACUC) of the RIKEN Yokohama Branch. Mice were maintained under specific pathogen-free (SPF) conditions in the animal facility at the RIKEN Yokohama Branch. 10-week-old male SPF (C57BL/6) mice were taken for experiment. Conditional *Piga* floxed mice were crossed with *villin-*Cre transgenic mice^67^ to obtain mice with intestinal epithelium-specific deletion of *Piga* gene. A total of six fresh fecal samples were collected from three *Piga* floxed mice (Control) and three intestinal epithelium-specific *Piga* gene knockout (*Piga*-/-) mice. The fecal samples were stored at − 80 °C before DNA extraction.

### DNA extraction

Fecal DNA extraction was performed as described previously^68^. Briefly, 10 mg of freeze-dried fecal samples were disrupted with 3 and 0.1 mm zirconia/silica beads by vigorous shaking (1,500 r.p.m. for 5 min) using a Shake Master (Biomedical Science) suspended in DNA extraction buffer containing 200 μL of 10% (w/v) SDS/TE (10 mM Tris–HCl, 1 mM EDTA, pH8.0) solution, 400 μL of phenol/chloroform/isoamyl alcohol (25:24:1), and 200 μL of 3 M sodium acetate. After centrifugation, bacterial genomic DNA was purified by the standard phenol/chloroform/isoamyl alcohol protocol. RNAs were removed from the sample by RNase A treatment.

### Whole metagenome shotgun sequencing (WMGS) and read quality improvement

The complete workflow of the metagenomic analysis is provided as S1 Fig. WMGS sequence libraries were developed using the Illumina TruSeq DNA Sample Preparation kit with catalog number PE-940–2001. Sequencing was carried out using the Illumina HiSeq2000 platform to produce paired-end reads of 125 bp. In a step of end repair, the fragments were purified using AMPureXP beads with gel-free method. The accuracy of raw reads was analysed using FastQC (https://www.bioinformatics.babraham.ac.uk/projects/fastqc/). The removal of the adapter sequences and the filtering of reads based on the Q-score (Q = 30) was performed using FaQCs (v1.34). Finally, the duplicated reads and the reads mapped on the host DNA were removed using PRINSEQ (PRINSEQ-lite 0.20.4) (http://prinseq.sourceforge.net/) and Bowtie2 (v2.2.5)^69^, respectively.

### Metagenomic data annotations and statistical analysis

The metagenomic data processing and analysis was done using the pipeline described previously^33^. Briefly, the high quality reads were aligned against the nr-db (as of 2017) with default parameters using the BLASTX option of DIAMOND (v0.9.9.110)^70^. The alignment files were then introduced into MEGAN6 (v6.8.18)^71^, where taxonomic and functional binning of the reads were carried out by selecting the Lowest Common Ancestor (LCA) algorithm with minimum bit score (50) and minimum support (50) parameters. The statistically significant differences between the control and *Piga-/-* mice metagenomic samples were identified using STAMP (v2.1.3)^72^ and Calypso^73^. The differences between these two groups were analyzed using Welch’s t-test with confidence interval 95% and p-value < 0.05. The taxonomic biomarkers between the groups were identified using Linear discriminant analysis Effect Size (LEfSe) analysis^74^. Alpha-diversity measures (on genus level) carried out using Shannon index and ANOVA (< 0.05) were used to determine the statistical significance among the two groups. In addition, on taxa belonging to dominant phylum F-test and T-test also applied to see the significant changes among the two groups. The statistically significant functions were backtraced on statistically significant taxa using EggNOG 4.5.1^75^.

### Animal study approval

All mice experiment procedures were approved by the Institutional Animal Care and Use Committee (IACUC) of the RIKEN Yokohama Branch and abide by all regulatory standards of IACUC of the RIKEN Yokohama Branch. We hereby confirm the study was carried out in compliance with the ARRIVE guidelines.

## Supplementary Information

Supplementary Information 1.

Supplementary Information 2.

Supplementary Information 3.

Supplementary Information 4.

Supplementary Information 5.

Supplementary Information 6.

Supplementary Information 7.

Supplementary Information 8.

## Data Availability

Metagenomic samples are available in the NCBI database with Bioproject ID PRJNA712972.
