# Supplementary material for: Gut microbiota alternation under the intestinal epithelium-specific knockout of mouse Piga gene
Source: Sci Rep. 2022 Jun 25;12:10812. doi: 10.1038/s41598-022-15150-5 (PMC9233684; doi:10.1038/s41598-022-15150-5)
Supplement: Supplementary file 3 — Supplementary Information 3. [file 41598_2022_15150_MOESM3_ESM.docx]

**S3 Table. List of significantly decreased taxa in the *Piga-/-* mice as compared to control and the roles they are known to play in physiologically important metabolites production.**

| **Taxa** | | | **SCFA production** | **p-value*** |
| --- | --- | --- | --- | --- |
| *Firmicutes; Clostridia; Clostridiales; Lachnospiraceae* | | |  |  |
| Genus | *Anaerocolumna* | |  | 0.018 |
|  | *Anaerosporobacter* | |  | 0.021 |
|  | *Bariatricus* | |  | 0.031 |
|  | *Eisenbergiella* | |  | 0.031 |
|  | *Lachnoclostridium* | | Butyrate [1] | 0.044 |
| Species | *Anaerosporobacter mobilis* | | Acetate [2] | 0.016 |
|  | *Bariatricus massiliensis* | | Butyrate [3] | 0.022 |
|  | *Eisenbergiella tayi* | | Butyrate, lactate, acetate and succinate [4] | 0.036 |
|  | *Clostridium symbiosum* | | Butyrate [5] | 0.020 |
|  | *Blautia schinkii* | |  | 0.021 |
|  | *Blautia obeum* | |  | 0.028 |
|  | *Blautia producta* | |  | 0.032 |
|  | *Clostridium clostridioforme* | | Butyrate [6] | 0.016 |
|  | *Clostridium bolteae* | | Acetate and lactate [7] | 0.019 |
|  | *Clostridium sp. KNHs209* | | Butyrate [8] | 0.037 |
|  | *Marvinbryantia formatexigens* | | Succinate, lactate and acetate [9] | 0.039 |
|  | *Ruminococcus torques* | | Butyrate [10] | 0.034 |
|  | *Roseburia hominis* | | Butyrate [11] | 0.027 |
|  | *Roseburia intestinalis* | | Butyrate [11] | 0.036 |
| *Firmicutes; Clostridia; Clostridiales; Ruminococcaceae* | | |  |  |
| Genus | | *Acetivibrio* |  | 0.038 |
| Species | | *Acetivibrio ethanolgignens* |  | 0.041 |
|  | | *Faecalibacterium prausnitzii* | Butyrate [11] | 0.046 |
|  | | *Ruminococcus gauvreauii* | Acetate [12] | 0.041 |
| *Firmicutes; Clostridia; Clostridiales; Clostridiaceae* | | |  |  |
| Genus | | *Hungatella* |  | 0.036 |
| Species | | *Hungatella hathewayi* | Propionate [13] | 0.020 |
|  | | *Lactonifactor longoviformis* |  | 0.020 |
| *Firmicutes; Clostridia; Clostridiales; Eubacteriaceae* | | |  |  |
| Species | | *Eubacterium ventriosum* | Butyrate [11] | 0.002 |
|  | | *Eubacterium plexicaudatum* | Butyrate [14] | 0.025 |

*p-value evaluated using Welch’s t-test.

**References**

1. Oliphant K, Allen-Vercoe E (2019) Macronutrient metabolism by the human gut microbiome: major fermentation by-products and their impact on host health. Microbiome 7: 91. doi: 10.1186/s40168-019-0704-8

2. Jeong H, Lim YW, Yi H, Sekiguchi Y, Kamagata Y, Chun J (2007) Anaerosporobacter mobilis gen. nov., sp. nov., isolated from forest soil. International journal of systematic and evolutionary microbiology 57: 1784-1787. doi: 10.1099/ijs.0.63283-0

3. Yuille S, Reichardt N, Panda S, Dunbar H, Mulder IE (2018) Human gut bacteria as potent class I histone deacetylase inhibitors in vitro through production of butyric acid and valeric acid. PloS one 13: e0201073. doi: 10.1371/journal.pone.0201073

4. Amir I, Bouvet P, Legeay C, Gophna U, Weinberger A (2014) Eisenbergiella tayi gen. nov., sp. nov., isolated from human blood. International journal of systematic and evolutionary microbiology 64: 907-914. doi: 10.1099/ijs.0.057331-0

5. Van den Abbeele P, Belzer C, Goossens M, Kleerebezem M, De Vos WM, Thas O, De Weirdt R, Kerckhof F-M, Van de Wiele T (2013) Butyrate-producing Clostridium cluster XIVa species specifically colonize mucins in an in vitro gut model. The ISME Journal 7: 949-961. doi: 10.1038/ismej.2012.158

6. Dehoux P, Marvaud JC, Abouelleil A, Earl AM, Lambert T, Dauga C (2016) Comparative genomics of Clostridium bolteae and Clostridium clostridioforme reveals species-specific genomic properties and numerous putative antibiotic resistance determinants. BMC genomics 17: 819. doi: 10.1186/s12864-016-3152-x

7. Song Y, Liu C, Molitoris DR, Tomzynski TJ, Lawson PA, Collins MD, Finegold SM (2003) Clostridium bolteae sp. nov., isolated from human sources. Syst Appl Microbiol 26: 84-89. doi: 10.1078/072320203322337353

8. Haas KN, Blanchard JL (2017) Kineothrix alysoides, gen. nov., sp. nov., a saccharolytic butyrate-producer within the family Lachnospiraceae. International journal of systematic and evolutionary microbiology 67: 402-410. doi: 10.1099/ijsem.0.001643

9. Ikeyama N, Sakamoto M, Ohkuma M, Hiramoto S, Wang J, Tone S, Shiiba K (2021) Fecal Microbiota Perspective for Evaluation of Prebiotic Potential of Bamboo Hemicellulose Hydrolysate in Mice: A Preliminary Study. Microorganisms 9. doi: 10.3390/microorganisms9050888

10. Kwak MS, Cha JM, Shin HP, Jeon JW, Yoon JY (2020) Development of a Novel Metagenomic Biomarker for Prediction of Upper Gastrointestinal Tract Involvement in Patients With Crohn’s Disease. Frontiers in Microbiology 11: 1162.

11. Vital M, Karch A, Pieper DH (2017) Colonic Butyrate-Producing Communities in Humans: an Overview Using Omics Data. mSystems 2. doi: 10.1128/mSystems.00130-17

12. Domingo MC, Huletsky A, Boissinot M, Bernard KA, Picard FJ, Bergeron MG (2008) Ruminococcus gauvreauii sp. nov., a glycopeptide-resistant species isolated from a human faecal specimen. International journal of systematic and evolutionary microbiology 58: 1393-1397. doi: 10.1099/ijs.0.65259-0

13. Ohara T (2019) Identification of the microbial diversity after fecal microbiota transplantation therapy for chronic intractable constipation using 16s rRNA amplicon sequencing. PloS one 14: e0214085-e0214085. doi: 10.1371/journal.pone.0214085

14. Bhat M, Pasini E, Copeland J, Angeli M, Husain S, Kumar D, Renner E, Teterina A, Allard J, Guttman DS, Humar A (2017) Impact of Immunosuppression on the Metagenomic Composition of the Intestinal Microbiome: a Systems Biology Approach to Post-Transplant Diabetes. Scientific Reports 7: 10277. doi: 10.1038/s41598-017-10471-2
