# Supplementary material for: Gut microbiota alternation under the intestinal epithelium-specific knockout of mouse Piga gene
Source: Sci Rep. 2022 Jun 25;12:10812. doi: 10.1038/s41598-022-15150-5 (PMC9233684; doi:10.1038/s41598-022-15150-5)
Supplement: Supplementary file 7 — Supplementary Information 7. [file 41598_2022_15150_MOESM7_ESM.docx]

**S7 Table. Details about functions of important intestine specific GPI-APs.**

| **Gene Name** | **Protein Name** | **Function** | **Location (Source: Uniprot)** |
| --- | --- | --- | --- |
| Gp2 | **Glycoprotein 2** | Expressed on the apical-surface of M cells and selectively binds FimH+ type I pili (present on multiple species of Gram-negative bacteria) [1]. | **Apical-surface of M cells [1].** |
| UMOD | **Uromodulin** | Closely related to GP2 [1].  Also shows binding towards the FimH+ Type 1 pili [1].  First found to be expressed on the apical surface of renal tubules epithelial cells and reported to shed into the urine where it binds to uropathogenic *E. coli* strain, interfering with bacterial adhesion and pathogenicity [1].  In M-cells, for *Lactobacillus acidophilus* UMOD serves as an uptake receptor [1]. | **Apical-surface of M cells [1].** |
| Prnp | **Major prion protein, PrP** | **PRNP also act like a uptake receptor via binding with conserved Hsp60 proteins expressed on multiple bacterial species along with *Brucella abortus* [1].** | **Apical-surface of M cells [1].** |
| Lypd8 | **Ly6/PLAUR domain-containing protein 8** | Secretory protein specifically needed to prevent gram-negative flagellated bacterial invasion in the inner-mucus layer [2]. | **Apical-surface of epithelial cells [2].** |
| Itgb1 | **Integrin β1** | **Acts as an uptake receptor for *Yersinia enterocolitica* [1].** | **Normally distributed in basolateral-surface of enterocytes and localizes in the apical-surface of M cells [1].** |
| Itln1a | **Intelectin-1a** (Galactofuranose-binding lectin) (Intestinal lactoferrin receptor) | In calcium dependent manner, this GPI-AP specifically recognizes microbial carbohydrate chains [3].  Binds to microbial glycans that contain a terminal acyclic 1,2-diol moiety, including D-phosphoglycerol-modified glycans, beta-linked D-galactofuranose, D-glycero-D-talo-oct-2-ulosonic acid and 3-deoxy-D-manno-oct-2-ulosonic acid.  Binds to glycans from both Gram-negative and Gram-positive bacteria including *K. pneumoniae*, *S. pneumoniae*, *Y. pestis*, *P. mirabilis* and *P. vulgaris*. However, does not show binding with mammalian glycans. Probably plays defense roles against microorganisms [3]. | Plasma membrane: Cell membrane; GPI-anchor  Extracellular region or Secreted: Secreted |
| Itln1b | **Intelectin-1b** | May play a protective role in the innate immune response to parasite infection [4]. | Plasma membrane: Cell membrane; GPI-anchor  Extracellular region or Secreted: Secreted |
| Iap | **Intestinal-type alkaline phosphatase, IAP, Intestinal alkaline phosphatase, EC 3.1.3.1** | Known to promote gut microbiota growth by decreasing the luminal concentration of nucleotide triphosphates [5].  Play role in local gut immunity maintenance [6]. | Plasma membrane: Cell membrane; GPI-anchor |
| Art2a | **T-cell ecto-ADP-ribosyltransferase 1, EC 2.4.2.31** | ART2a is needed for cell surface proteins ADP-ribosylation [7].  GPI-AP linking paracrine/autocrine activation of inflammatory macrophages to the release of nicotinamide adenine dinucleotide (NAD) (a critical intracellular metabolite) [8].  Extracellular NAD acts via ART2.1 and does ADP ribosylation to P2X7 purinergic receptor. Play important roles in inflammatory responses in murine macrophages [9]. | Plasma membrane: Cell membrane; GPI-anchor |
| Art2b | **T-cell ecto-ADP-ribosyltransferase 2, EC 2.4.2.31** | Has both NAD^+^ glycohydrolase and ADP-ribosyltransferase activity [10, 11]. | Plasma membrane: Cell membrane; GPI-anchor |
| **Mill1** | **MHC class I-like protein MILL1** (MHC class I-like located near the leukocyte receptor complex 1) | Lacks key residues involved in peptide docking and also does not require TAP (transporter involved in antigen processing) for cell surface expression, suggesting that this is a non-classical MHC class I protein which does not play a role in antigen presentation [12, 13]. | Plasma membrane: Cell membrane; GPI-anchor |
| Fcgr4 | **Low affinity immunoglobulin gamma Fc region receptor IV** | Receptor for the Fc region of immunoglobulin gamma [14].  Binding to IgE promotes macrophage-mediated phagocytosis, antigen presentation to T cells, production of proinflammatory cytokines and the late phase of cutaneous allergic reactions [15, 16]. | Plasma membrane: Cell membrane, single pass type-I membrane protein |
| **Function predicted based on similarity:** Source Uniprot Database | | |  |
| Xpnpep2 | **Xaa-Pro aminopeptidase 2, EC 3.4.11.9** | Membrane-bound metalloprotease which catalyzes the removal of a penultimate prolyl residue from the N-termini of peptides, such as Arg-Pro-Pro.  May play a role in the metabolism of the vasodilator bradykinin. | Plasma membrane: Cell membrane; GPI-anchor |
| Prss30 | **Serine protease 30, EC 3.4.21.-** (Distal intestinal serine protease) (Transmembrane serine protease 8) | Selectively cut on synthetic-peptide substrates of trypsin and activates the epithelial sodium ion channel (ENaC). | Plasma membrane: Cell membrane; GPI-anchor |
| Gpc6 | **Glypican-6** [Cleaved into: **Secreted glypican-6**] | Cell surface proteoglycan that bears heparan sulfate. Putative cell surface coreceptor for growth factors, extracellular matrix proteins, proteases and anti-proteases. Enhances migration and invasion of cancer cells through WNT5A signaling. | Plasma membrane: Cell membrane; Extracellular side; GPI-anchor  Extracellular region or Secreted: extracellular space |
| Treh | **Trehalase, EC 3.2.1.28** (Alpha,alpha-trehalase) (Alpha,alpha-trehalose glucohydrolase) | Intestinal trehalase is probably involved in the hydrolysis of ingested trehalose. | Plasma membrane: Cell membrane; GPI-anchor |
| Ly6m 2010109I03Rik | Lymphocyte antigen 6 complex, locus M | acetylcholine receptor inhibitor activity. | Plasma membrane: Plasma membrane  Other locations: anchored component of membrane |

**References**

1. Kanaya T, Williams IR, Ohno H (2020) Intestinal M cells: Tireless samplers of enteric microbiota. Traffic 21: 34-44. doi: 10.1111/tra.12707

2. Okumura R, Kurakawa T, Nakano T, Kayama H, Kinoshita M, Motooka D, Gotoh K, Kimura T, Kamiyama N, Kusu T, Ueda Y, Wu H, Iijima H, Barman S, Osawa H, Matsuno H, Nishimura J, Ohba Y, Nakamura S, Iida T, Yamamoto M, Umemoto E, Sano K, Takeda K (2016) Lypd8 promotes the segregation of flagellated microbiota and colonic epithelia. Nature 532: 117-121. doi: 10.1038/nature17406

3. Wesener DA, Wangkanont K, McBride R, Song X, Kraft MB, Hodges HL, Zarling LC, Splain RA, Smith DF, Cummings RD, Paulson JC, Forest KT, Kiessling LL (2015) Recognition of microbial glycans by human intelectin-1. Nat Struct Mol Biol 22: 603-610. doi: 10.1038/nsmb.3053

4. Pemberton AD, Knight PA, Wright SH, Miller HR (2004) Proteomic analysis of mouse jejunal epithelium and its response to infection with the intestinal nematode, Trichinella spiralis. Proteomics 4: 1101-1108. doi: 10.1002/pmic.200300658

5. Malo MS, Moaven O, Muhammad N, Biswas B, Alam SN, Economopoulos KP, Gul SS, Hamarneh SR, Malo NS, Teshager A, Mohamed MM, Tao Q, Narisawa S, Millán JL, Hohmann EL, Warren HS, Robson SC, Hodin RA (2014) Intestinal alkaline phosphatase promotes gut bacterial growth by reducing the concentration of luminal nucleotide triphosphates. Am J Physiol Gastrointest Liver Physiol 306: G826-838. doi: 10.1152/ajpgi.00357.2013

6. Chen KT, Malo MS, Beasley-Topliffe LK, Poelstra K, Millan JL, Mostafa G, Alam SN, Ramasamy S, Warren HS, Hohmann EL, Hodin RA (2011) A role for intestinal alkaline phosphatase in the maintenance of local gut immunity. Dig Dis Sci 56: 1020-1027. doi: 10.1007/s10620-010-1396-x

7. Ohlrogge W, Haag F, Löhler J, Seman M, Littman DR, Killeen N, Koch-Nolte F (2002) Generation and characterization of ecto-ADP-ribosyltransferase ART2.1/ART2.2-deficient mice. Mol Cell Biol 22: 7535-7542. doi: 10.1128/mcb.22.21.7535-7542.2002

8. Hong S, Brass A, Seman M, Haag F, Koch-Nolte F, Dubyak GR (2007) Lipopolysaccharide, IFN-gamma, and IFN-beta induce expression of the thiol-sensitive ART2.1 Ecto-ADP-ribosyltransferase in murine macrophages. J Immunol 179: 6215-6227. doi: 10.4049/jimmunol.179.9.6215

9. Hong S, Schwarz N, Brass A, Seman M, Haag F, Koch-Nolte F, Schilling WP, Dubyak GR (2009) Differential regulation of P2X7 receptor activation by extracellular nicotinamide adenine dinucleotide and ecto-ADP-ribosyltransferases in murine macrophages and T cells. J Immunol 183: 578-592. doi: 10.4049/jimmunol.0900120

10. Kanaitsuka T, Bortell R, Stevens LA, Moss J, Sardinha D, Rajan TV, Zipris D, Mordes JP, Greiner DL, Rossini AA (1997) Expression in BALB/c and C57BL/6 mice of Rt6-1 and Rt6-2 ADP-ribosyltransferases that differ in enzymatic activity: C57BL/6 Rt6-1 is a natural transferase knockout. J Immunol 159: 2741-2749.

11. Adriouch S, Bannas P, Schwarz N, Fliegert R, Guse AH, Seman M, Haag F, Koch-Nolte F (2008) ADP-ribosylation at R125 gates the P2X7 ion channel by presenting a covalent ligand to its nucleotide binding site. Faseb j 22: 861-869. doi: 10.1096/fj.07-9294com

12. Kasahara M, Watanabe Y, Sumasu M, Nagata T (2002) A family of MHC class I-like genes located in the vicinity of the mouse leukocyte receptor complex. Proc Natl Acad Sci U S A 99: 13687-13692. doi: 10.1073/pnas.212375299

13. Kajikawa M, Baba T, Tomaru U, Watanabe Y, Koganei S, Tsuji-Kawahara S, Matsumoto N, Yamamoto K, Miyazawa M, Maenaka K, Ishizu A, Kasahara M (2006) MHC class I-like MILL molecules are beta2-microglobulin-associated, GPI-anchored glycoproteins that do not require TAP for cell surface expression. J Immunol 177: 3108-3115. doi: 10.4049/jimmunol.177.5.3108

14. Nimmerjahn F, Bruhns P, Horiuchi K, Ravetch JV (2005) FcgammaRIV: a novel FcR with distinct IgG subclass specificity. Immunity 23: 41-51. doi: 10.1016/j.immuni.2005.05.010

15. Hirano M, Davis RS, Fine WD, Nakamura S, Shimizu K, Yagi H, Kato K, Stephan RP, Cooper MD (2007) IgEb immune complexes activate macrophages through FcgammaRIV binding. Nature immunology 8: 762-771. doi: 10.1038/ni1477

16. Mancardi DA, Iannascoli B, Hoos S, England P, Daëron M, Bruhns P (2008) FcgammaRIV is a mouse IgE receptor that resembles macrophage FcepsilonRI in humans and promotes IgE-induced lung inflammation. J Clin Invest 118: 3738-3750. doi: 10.1172/jci36452
