# Supplementary material for: Gut microbiota alternation under the intestinal epithelium-specific knockout of mouse Piga gene
Source: Sci Rep. 2022 Jun 25;12:10812. doi: 10.1038/s41598-022-15150-5 (PMC9233684; doi:10.1038/s41598-022-15150-5)
Supplement: Supplementary file 8 — Supplementary Information 8. [file 41598_2022_15150_MOESM8_ESM.docx]

**Gut microbiota alternation under the intestinal epithelium-specific knockout of mouse *Piga* gene.**

**Running Title: Gut-microbiota dynamics in *Piga* deficient mice.**

Aditi Jangid^1^, Shinji Fukuda^2-5^, Masahide Seki^6^, Yutaka Suzuki^6^, Todd D Taylor^7^, Hiroshi Ohno^3,4^, Tulika Prakash^1,7^ *

^1^ BioX Centre and School of Basic Sciences, Indian Institute of Technology Mandi, Kamand, Mandi, Himachal Pradesh – 175005, India.

^2^ Institute for Advanced Biosciences, Keio University, Tsuruoka, Yamagata – 997-0052, Japan.

^3^ Laboratory for Intestinal Ecosystem, RIKEN Center for Integrative Medical Sciences, Yokohama, Kanagawa – 230-0045, Japan.

^4^ Intestinal Microbiota Project, Kanagawa Institute of Industrial Science and Technology, Kawasaki, Kanagawa- 210-0821, Japan.

^5^ Transborder Medical Research Center, University of Tsukuba, Tsukuba, Ibaraki – 305-8575, Japan.

^6^ Department of Computational Biology and Medical Sciences, The University of Tokyo 5-1-5, Kashiwanoha, Kashiwa Chiba – 277-8562, Japan.

^7^ Laboratory for Microbiome Sciences, RIKEN Center for Integrative Medical Sciences, Tsurumi-ku, Yokohama, Kanagawa – 230-0045, Japan.

* Corresponding Author

Email: tulika@iitmandi.ac.in

**
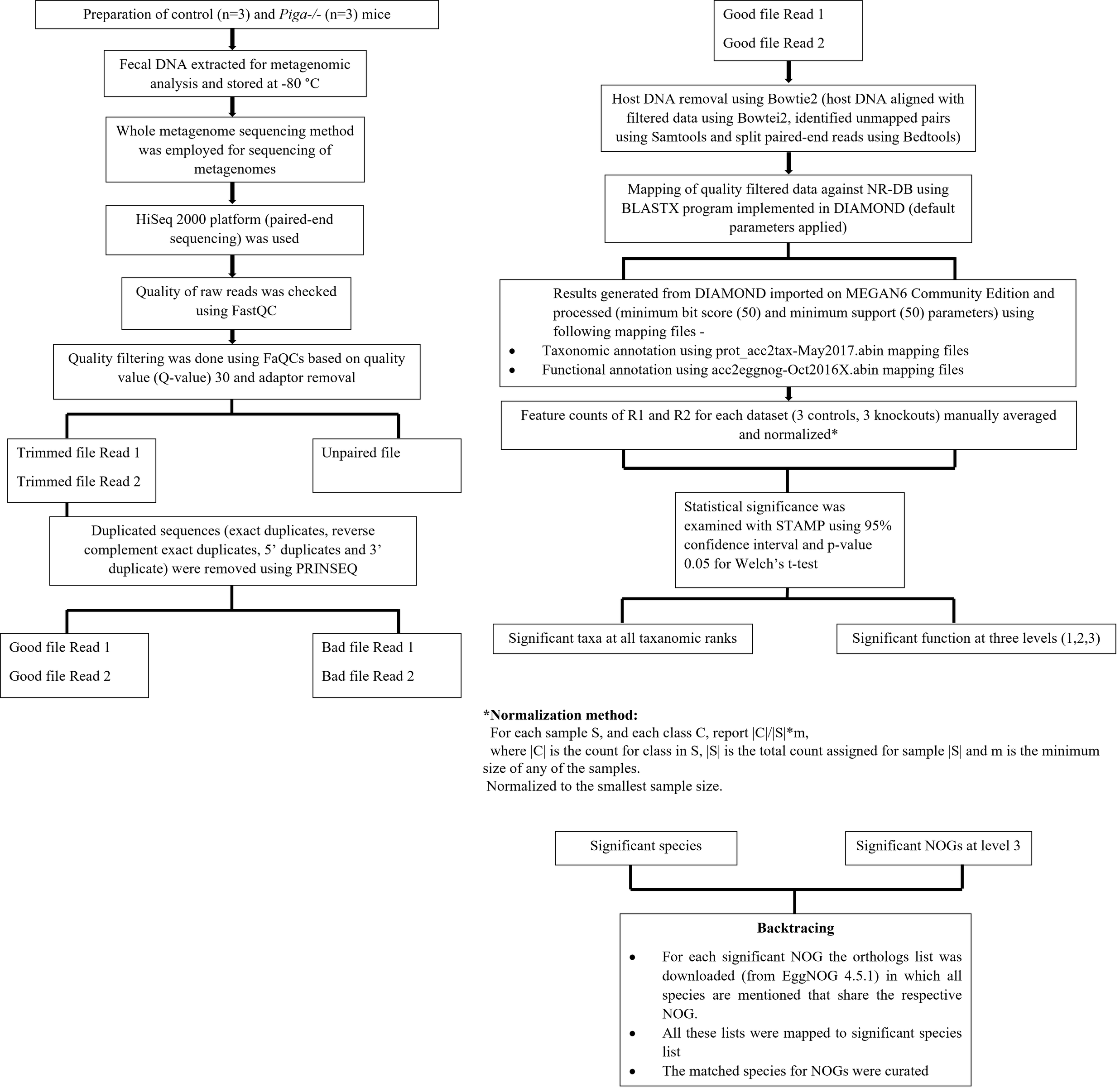
**

**S1 Fig. Workflow for whole metagenome shotgun sequencing data processing and analysis.**

**
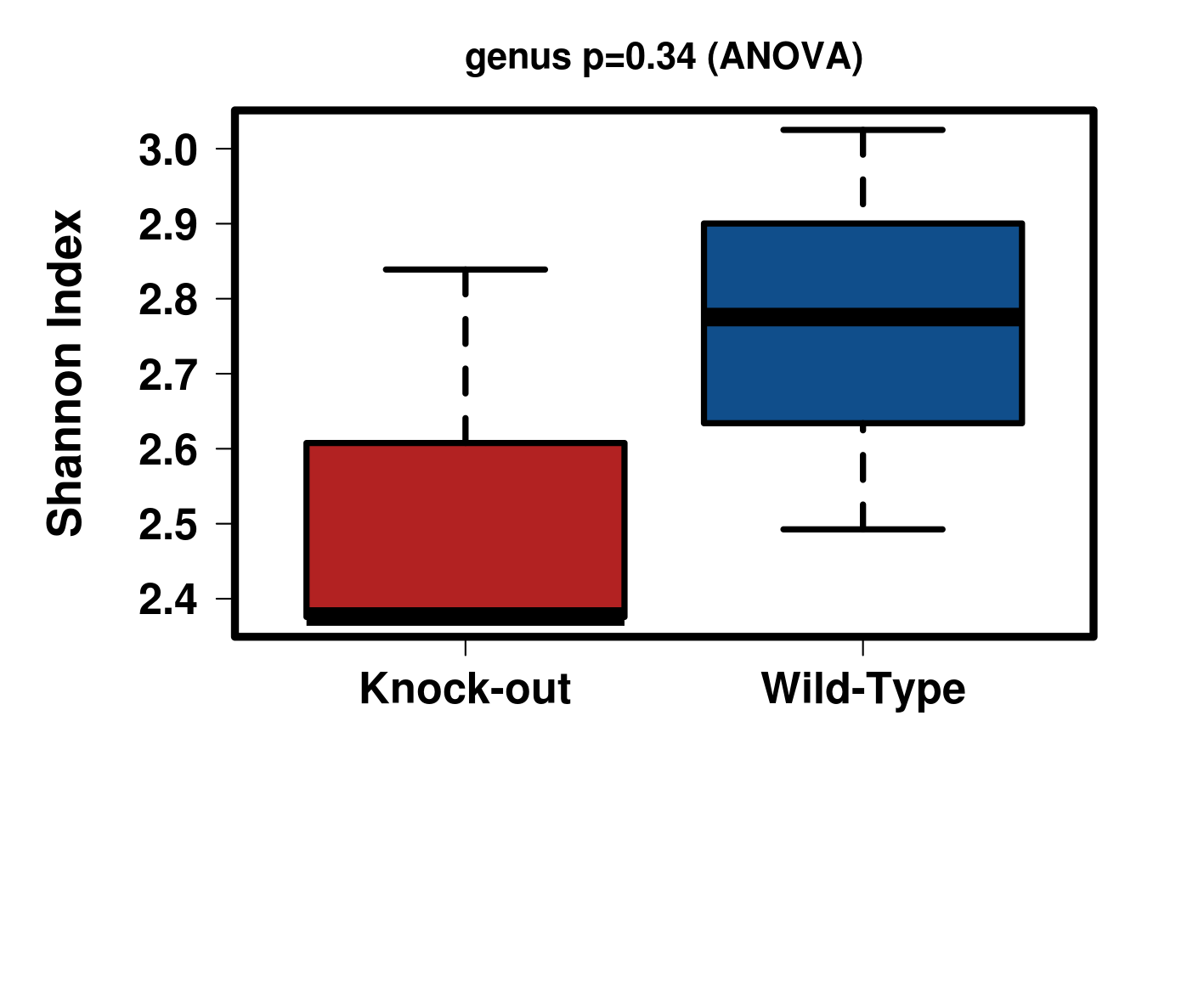
**

**S2 Fig. Alpha diversity measures among *Piga-/-* and control mice.**

**
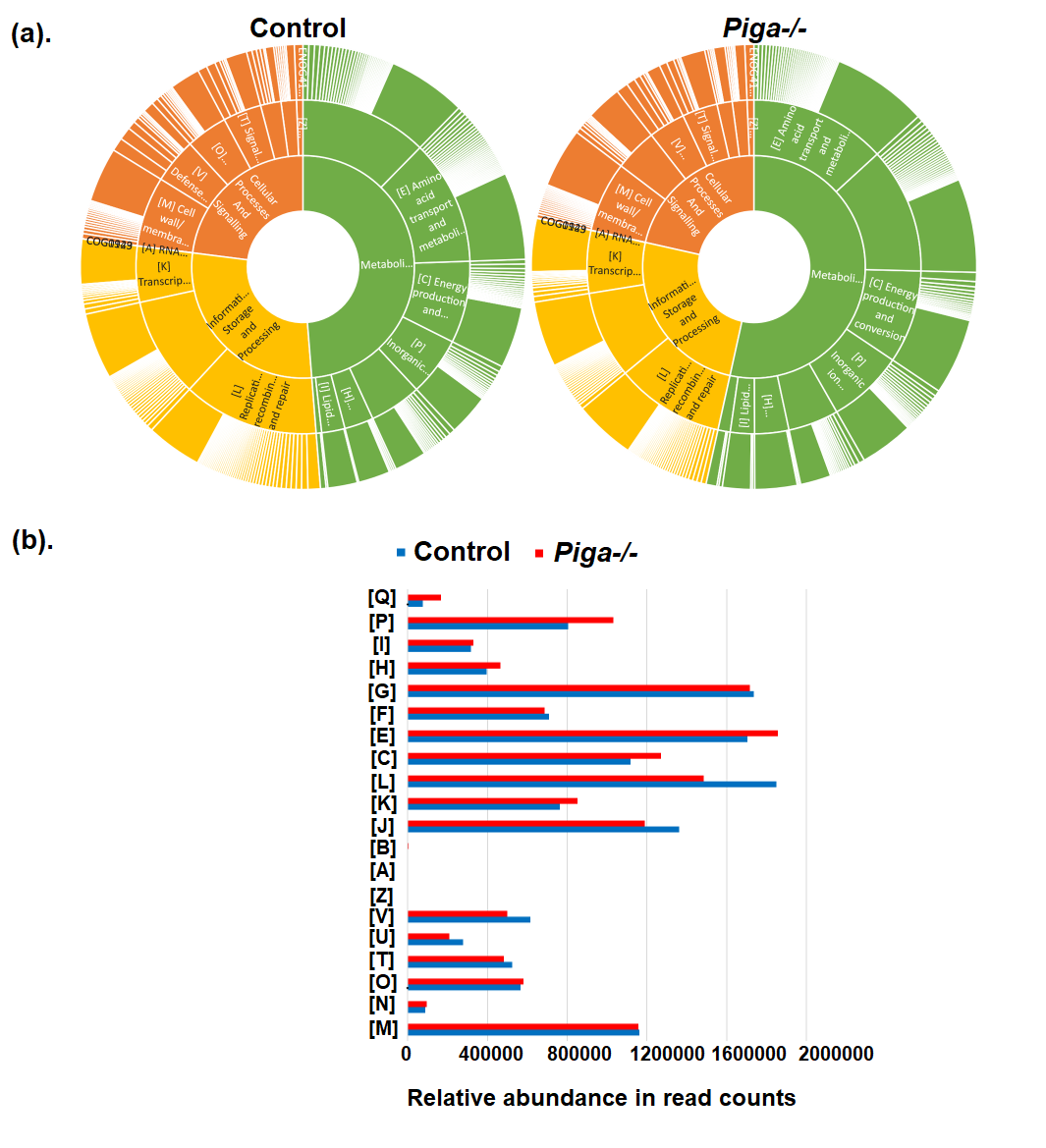
**

**S3 Fig. Relative abundances of predicted functions in the *Piga-/-* and control samples shown on EggNOG based classifications.** (a) Sunburst chart representing the relative abundances at three functional hierarchical levels, namely, level-1 (innermost), level-2 (middle), and level-3 (outermost). (b) Bar plot representing the relative abundance at level-2. [B] Chromatin structure and dynamics; [C] Energy production and conversion; [D] Cell cycle control, cell division, chromsome partitioning; [E] Amino acid transport and metabolism; [F] Nucleotide transport and metabolism; [G] Carbohydrate transport and metabolism; [H] Coenzyme transport and metabolism; [I] Lipid transport and metabolism; [J] Translation, ribosomal structure and biogenesis; [K] Transcription; [L] Replication, recombination and repair; [M] Cell wall/membrane/envelope biogenesis; [N] Cell motility; [O] Posttranslational modification, protein turnover, chaperones; [P] Inorganic ion transport and metabolism; [Q] Secondary metabolites biosynthesis, transport and catabolism; [T] Signal Transduction mechanisms; [U] Intracellular trafficking, secretion, and vesicular transport; [V] Defense mechanisms.

**
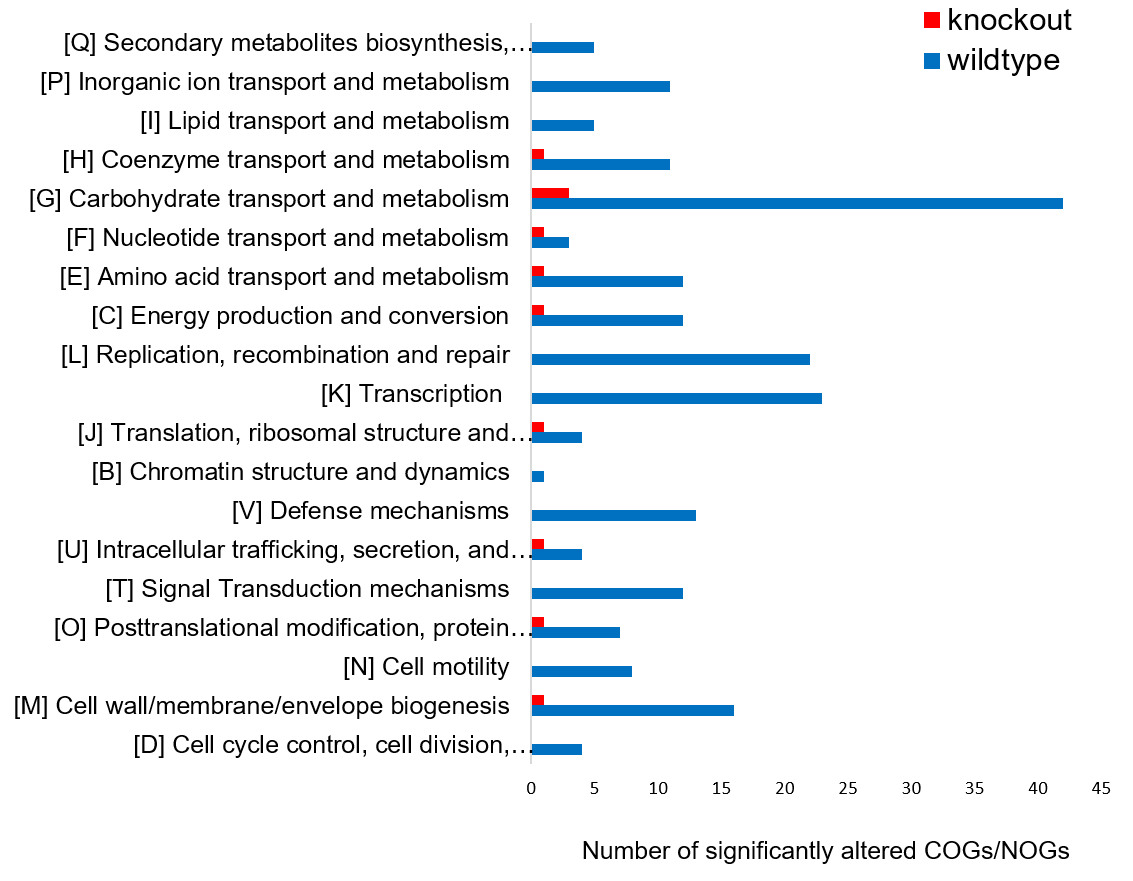
**

**S4 Fig. Bar plot representing the numbers of significantly altered functions in the *Piga-/-* and control groups.** A total of 226 COGs/NOGs were found to be significantly altered between the control and *Piga-/-* groups at level -3. The bar plot represents the distribution of these 226 COGs/NOGs in the *Piga-/-* and control groups.
